# Transcriptomics, Cheminformatics, and Systems Pharmacology Strategies Unveil the Potential Bioactives to Combat COVID-19

**DOI:** 10.3390/molecules27185955

**Published:** 2022-09-13

**Authors:** Sivakumar Adarshan, Sakthivel Akassh, Krishnakumar Avinash, Mathivanan Bharathkumar, Pandiyan Muthuramalingam, Hyunsuk Shin, Venkidasamy Baskar, Jen-Tsung Chen, Veluswamy Bhuvaneshwari, Manikandan Ramesh

**Affiliations:** 1 Department of Biotechnology, Science Campus, Alagappa University, Karaikudi 630003, India; 2 Department of Biotechnology, Sri Shakthi Institute of Engineering and Technology, Coimbatore 641062, India; 3 Division of Horticultural Science, College of Agriculture and Life Sciences, Gyeongsang National University, Jinju 52725, Korea; 4 Department of GreenBio Science, Gyeongsang National University, Jinju 52725, Korea; 5 Department of Oral and Maxillofacial Surgery, Saveetha Dental College and Hospitals, Saveetha Institute of Medical and Technical Sciences, Chennai 602105, India; 6 Department of Life Sciences, National University of Kaohsiung, Kaohsiung 811, Taiwan; 7 Department of Biotechnology, PSGR Krishnammal College for Women, Coimbatore 641004, India

**Keywords:** COVID-19, cheminformatics, molecular docking, phytocompounds, systems pharmacology

## Abstract

Coronavirus disease (COVID-19) is a viral disease caused by the SARS-CoV-2 virus and is becoming a global threat again because of the higher transmission rate and lack of proper therapeutics as well as the rapid mutations in the genetic pattern of SARS-CoV-2. Despite vaccinations, the prevalence and recurrence of this infection are still on the rise, which urges the identification of potential global therapeutics for a complete cure. Plant-based alternative medicine is becoming popular worldwide because of its higher efficiency and minimal side effects. Yet, identifying the potential medicinal plants and formulating a plant-based medicine is still a bottleneck. Hence, in this study, the systems pharmacology, transcriptomics, and cheminformatics approaches were employed to uncover the multi-targeted mechanisms and to screen the potential phytocompounds from significant medicinal plants to treat COVID-19. These approaches have identified 30 unique COVID-19 human immune genes targeted by the 25 phytocompounds present in four selected ethnobotanical plants. Differential and co-expression profiling and pathway enrichment analyses delineate the molecular signaling and immune functional regulations of the COVID-19 unique genes. In addition, the credibility of these compounds was analyzed by the pharmacological features. The current holistic finding is the first to explore whether the identified potential bioactives could reform into a drug candidate to treat COVID-19. Furthermore, the molecular docking analysis was employed to identify the important bioactive compounds; thus, an ultimately significant medicinal plant was also determined. However, further laboratory evaluation and clinical validation are required to determine the efficiency of a therapeutic formulation against COVID-19.

## 1. Introduction

Coronavirus disease (COVID-19), a contagious viral disease that emerged as a pandemic in 2020, is still a global threat because of the higher transmission rate. The causative agent for this disease is the SARS-CoV-2 virus, which belongs to the family of Coronaviridae [1]. Dry cough, pneumonia, fever, and fatigue are the common symptoms of this disease [2]. COVID disease is frequently associated with a lung infection and respiratory illness, which are the hallmarks of this disease [3]. COVID-19 has become a deadly world health crisis. Presently, the global prevalence of this disease has crossed 603 million cases with more than 6.4 million deaths (WHO—https://covid19.who.int/; https://www.cgtn.com/special/The-latest-on-the-COVID-19-pandemic.html, accessed on 7 September 2022) [4]. Vaccines play a major role in preventing this disease, but re-infection of this disease is still prevalent [5,6]. Despite the advanced medicinal technologies and facilities, a complete cure for this disease is still not identified. Furthermore, targeting the viral proteins is a major bottleneck due to the rapid mutations of the virus. Hence, identification of the human targets and novel therapeutic drugs are needed immediately.

Medicinal plants comprise plenty of biologically active pharmaceutical agents, most significantly in the era of disease treatments [7]. Medicinal plants have long been considered a massive storehouse of potent resources for humankind [1,8,9,10]. Exploring plant bioactive molecules could be a potential strategy to identify new therapeutic molecules, including anti-viral drugs, due to their structural and chemical diversity. Furthermore, plant bioactives have been utilized for pharmacotherapeutic purposes around the globe for a long time [1,9,11]. Some traditional Indian medicinal plants such as *Ammi visnaga*, *Amaranthus viridis*, *Terminalia bellirica,* and *Piper betle* were used to combat respiratory tract diseases [12,13,14,15]. Furthermore, these medicinal plants have diverse pharmacological activities such as immune-modulator, anti-viral, anti-microbial, anti-inflammatory, anti-tumorigenic, anti-cancer (breast, ovarian, cervical cancer), anti-mutagenic, antioxidant, analgesic, and anti-depressant [12,13,14,15].

One unique way to learn in-depth about how active biomolecules perform their therapeutic function is to impute the gene networks regulated by medicinal plant bioactive molecules [16,17]. In drug discovery, the one drug–one target–one disease strategy has become highly inefficient. Though single target methods might be a useful approach for single gene disorders, for multiple-gene-associated diseases, such one-target strategies are not fruitful [16]. Therefore, the concept of developing a multi-target treatment strategy against complex diseases, including COVID-19, COPD, and Alzheimer’s diseases, is emerging in drug discovery. Regarding this, systems pharmacology unravels the disease mechanisms as potential networks targeted by synergistic, multiple drugs. Recently, the use of network/systems pharmacology for an in-depth understanding of the mode of action of bioactive compounds has become the most popular [1,9,10].

Considering the importance of Indian traditional medicine and network pharmacology, how the bioactive molecules will act and what all of their essential human targets are still remain major roadblocks. The main aim of this holistic study is to explore the prominent molecular mechanism and pharmaceutical activities of bioactive molecules and important medicinal plants against COVID-19. In addition, emerging advancements in systems pharmacology, transcriptomics, and important analytical tools such as cheminformatics and molecular docking analyses have paved the way to understanding the molecular mechanism of Indian traditional medicine to combat this deadly disease. Hence, the present study focuses on the transcriptomics, cheminformatics, molecular docking, and systems pharmacology approaches to identify the differentially expressed COVID-19-associated genes and the potential bioactive compounds from crucial medicinal plants such as *A. visnaga*, *A. viridis*, *T. bellirica,* and *P. betle*, which can be employed in the formulation of a cure from COVID-19, after clinical validation. This study would serve as an essential tool for dissecting novel biomolecules and their importance in traditionally used medicinal plants.

## 2. Materials and Methods

### 2.1. Selection of Medicinal Plants

Medicinal plants that are easily available in abundance, ethnobotanically important, and commonly utilized in Indian households have been selected, which include *A. visnaga, A. viridis, T. bellirica,* and *P. betle* [12,13,14,15].

### 2.2. Retrieval of Phytocompounds

Phytocompounds are the bioactive compounds present in plants with the characteristics of therapeutic potential. Phytocompounds present in the selected medicinal plants were identified using a literature survey and web sources [12,13,14,15]. The canonical SMILES for those phytocompounds were collected from the PubChem database (https://pubchem.ncbi.nlm.nih.gov/, accessed on 23 February 2022) [18].

### 2.3. Mining of Human Targets

Canonical SMILES obtained from the PubChem database were used as an input to obtain the compound-specific human targets through the SwissTargetPrediction tool (www.swisstargetprediction.ch/, accessed on 23 February 2022) [19]. In addition, reported 130 SARS-CoV-2-associated genes (Supplementary Table S1) were collected [1,20,21,22] for manual comparative analysis to find the unique genes.

### 2.4. Over-Representation Analysis (ORA)

ORA analysis is one of the most common statistical methods for determining over-represented genes in the subset of obtained data [23]. Collated receptors were provided as the input onto Network Analyst (www.networkanalyst.ca/, accessed on 12 March 2022) to identify the involvement of these genes in various molecular activities [24].

### 2.5. Compound-Target-Network (C-T-N)

C-T-N was constructed using the Cytoscape v3.8.2 plugin [25], which helps to investigate the deeper mechanisms of phytocompounds’ activity. This network helps to understand the multiple interacting partners of the phytocompounds, thus providing the collective potentials of those compounds [26]. Furthermore, the understanding of the interactions between the potential target genes was then visualized by GeneMANIA (https://genemania.org/, accessed on 12 March 2022) [27].

### 2.6. Analyses of Whole Blood Human Transcriptome of Healthy Control and SARS-CoV-2 Infection

The identified unique genes were imported to COVID19db (http://hpcc.siat.ac.cn/covid19db/home, accessed on 2 September 2022) [28] and a complete method was used to obtain the differential expression heatmap plot against the human COVID-19 transcriptomic datasets of GEO (Gene Expression Omnibus) accession of healthy controls (GSM4622633, GSM4622634, GSM4622635, GSM4622636, GSM4622637) and COVID-19-infected (GSM4622702, GSM4622703, GSM4622704, GSM4622705, GSM4622706) whole blood tissue. Further, GO (Gene Ontology) enrichment analysis of these unique COVID-19-associated genes was also performed against these datasets in the COVID19db differential expression module [28] with significant parameter threshold values, such as log2FC cutoff ≥ 1, *p*-value cutoff ≤ 0.05, dysregulation type—all, GO-*p*-value cutoff ≤ 0.1, and GO-*q*-value cutoff ≤ 0.3 to attain the GO biological process and molecular functions according to the enriched GO terms.

### 2.7. Pathway Enrichment Analysis of Unique COVID-19 Genes

Biological pathways enrichment (Kyoto Encyclopedia of Genes and Genomes-KEGG) analysis of COVID-19-associated unique genes was carried out through g:Profiler (https://biit.cs.ut.ee/gprofiler/gost, accessed on 4 September 2022) [29] against “Homo sapiens” and a KEGG term ID with an adjusted *p*-value < 0.05 was considered as significant. The KEGG pathway terms with adjusted *p*-values were supposed to have potent effects on combating deadly COVID-19, and the pathway terms were illustrated by an adjusted *p*-value from low to high.

### 2.8. Corrplot Analysis

The unique COVID-19-responsible genes were uploaded to the corrplot co-expression module of COVID19db (http://hpcc.siat.ac.cn/covid19db/home, accessed on 2 September 2022) [28] against the above-mentioned COVID-19 transcriptomic datasets of GEO accession of healthy controls and COVID-19-infected whole blood tissue was employed with a circle visualization method to understand the correlationships among each two players. Further, a *p*-value cutoff was to set as a threshold for the significant correlation in accordance with the inbuilt database statistical program.

### 2.9. Box and Volcano Plot Analyses

The maximum phytocompound-linked and crucial COVID-19-pathway-associated unique genes were selected manually and subjected to the box plot differential expression module of COVID19db (http://hpcc.siat.ac.cn/covid19db/home, accessed on 2 September 2022) [28] against the above-mentioned GEO accession of healthy controls and COVID-19-infected whole blood tissue, executed with an inbuilt ANOVA statistical method to understand the expression of the players involved. Similarly, volcano plot analysis was also employed in the COVID19db [28] expression module with two significant parameters, such as log2FC and *p*-value cutoffs of ≥ 1 and ≤ 0.05, respectively, to unveil the players’ expression level.

### 2.10. Active Compound Property

The Molinspiration tool (https://www.molinspiration.com/, accessed on 5 April 2022) [30] was employed to identify the features of these compounds, including a number of violations (nVio), enzyme inhibitory activity (Ei), Kinase inhibitory activity (Ki), Protease inhibitory activity (Pi), GPCR ligand activity (GPCR), and enzymes and nuclease receptors (Ncr). Among these features, nVio is the significant property in determining the credibility of using these compounds as a potent drug [1,9].

### 2.11. Molecular Docking

COVID-19-associated genes identified through differential expression profiling and their respective proteins’ 3D structures were retrieved from PDB (protein data bank), and further energy was minimized by chimera [31]. Finally, molecular docking was performed for the compounds with their respective ligands using Autodock Vina 1.1 [32] to identify the potential compound that can be employed in the treatment of COVID-19 disease.

### 2.12. Toxicity and Drug Likeliness Properties

The potential phytocompounds identified using molecular docking were further subjected to the toxicity prediction analysis. AdmetSAR (http://lmmd.ecust.edu.cn/admetsar2/, accessed on 4 September 2022) [33] and SwissADME (http://www.swissadme.ch/, accessed on 4 September 2022) [34] online tools were employed to screen the toxic properties and health effects on human organs [35]. In addition, the drug likeliness properties of these compounds were analyzed using the SwissADME tool.

## 3. Results

### 3.1. Collation of Phytocompounds

A total of 34 phytocompounds were scrutinized from the selected medicinal plants, such as *A. visnaga, A. viridis, T. bellirica*, and *P. betle* (Table 1). The canonical SMILES and the structure for the selected phytocompounds are provided in Supplementary Table S2.

### 3.2. Human Targets and Unique COVID-Associated Genes Mining

Phytocompounds targeting human receptors were identified through SwissTargetPrediction. Study results revealed that out of 34 selected phytocompounds, 25 compounds targeting 375 human targets (9 compounds, namely AMM, KLOL, GALLO, CHAVIA, CHAVIME, CAPE, FPIN, LIME, and SPRO were eliminated from the further studies since these compounds do not have potential binding targets). Based on the gene probability score, genes are selected and shown in Table 2, which lists the gene features for each of the compounds along with the Uniprot ID, chromosome number, and orthologous information of the target. For identifying the unique COVID-19-associated genes obtained by the human targets and already reported 130 COVID-19-associated genes were matched and identified the 30 common/unique genes and the list of all the compounds with their unique active targets is provided in the Supplementary Table S3. It was observed that the 25 compounds were notably targeting these 30 unique COVID-19-associated genes.

### 3.3. ORA Enrichment

**Figure 1:**
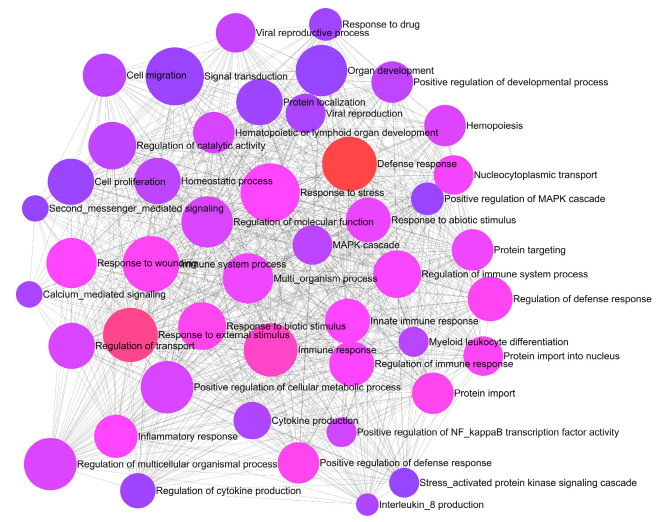
ORA predicts the involvement of genes in various activities.

ORA analysis using the Network Analyst tool predicted the involvement of these genes in numerous important pathways, such as the regulation of cytokine production, interleukin 8 production, and the viral reproductive process, which are important for the regulation of viruses (Figure 1). The activity of the selected phytocompounds on these targets may lessen the risk and severity of COVID-19 infection.

### 3.4. Compound-Target-Network Analysis

**Figure 2:**
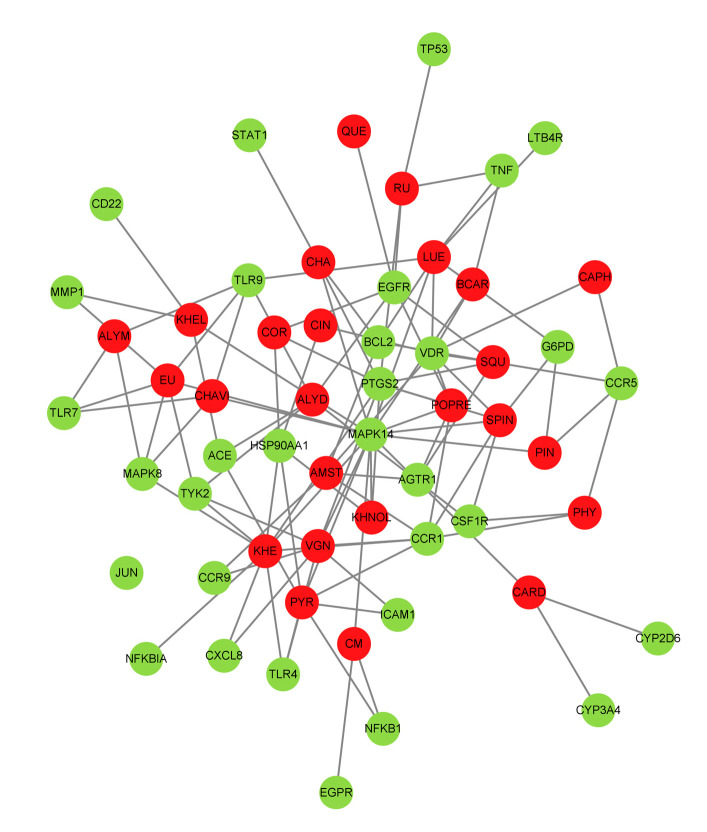
Compound-target-network (C-T-N) analysis depicts the interaction between the phytocompounds and human COVID-19 immune targets. Green color represents human COVID-19 immune targets and the red color indicates compounds.

The C-T-N analysis was performed using Cytoscape v3.8.2 to visualize the interactions of 25 compounds targeting their 30 unique COVID-19-linked human targets (Figure 2). This interaction analysis helps to understand the multiple-target potentiality of these compounds and justifies the activity of these compounds as a potent drug to treat COVID-19.

### 3.5. Cross-Talks of Genes

**Figure 3:**
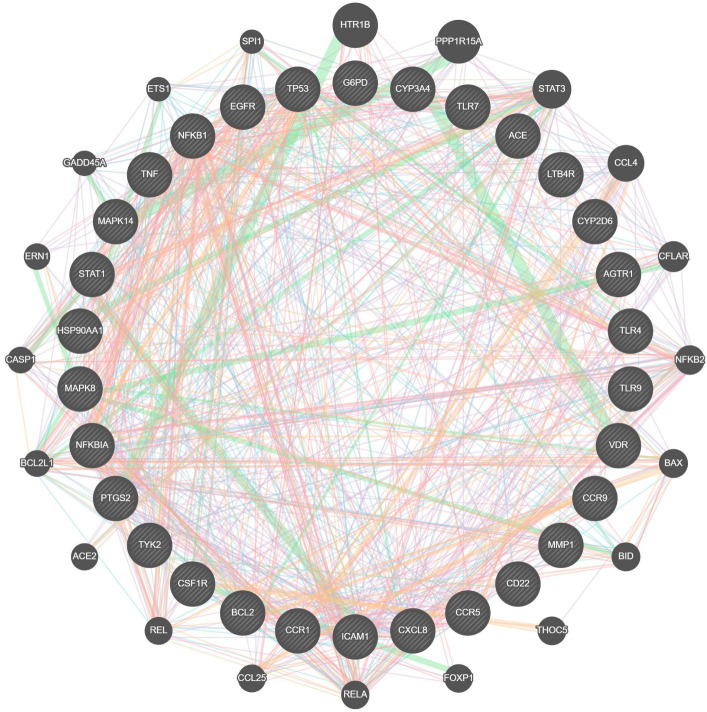
Visualization of COVID-19-associated unique gene interaction. Colored lines between the genes indicate various types of interacting evidence: periwinkle—co-expression; orange—physical interaction; green—genetic interaction; blue—localization; sky blue—involvement in pathways.

Molecular interactions between 30 potential target genes revealed the complex interactions between the target genes and were visualized by GeneMANIA (Figure 3). These signaling cross-talks represent various types of interacting evidence, such as co-expression, and physical and genetic interactions.

### 3.6. Transcriptomic and Enrichment Profiling of Unique COVID-19-Associated Genes

**Figure 4:**
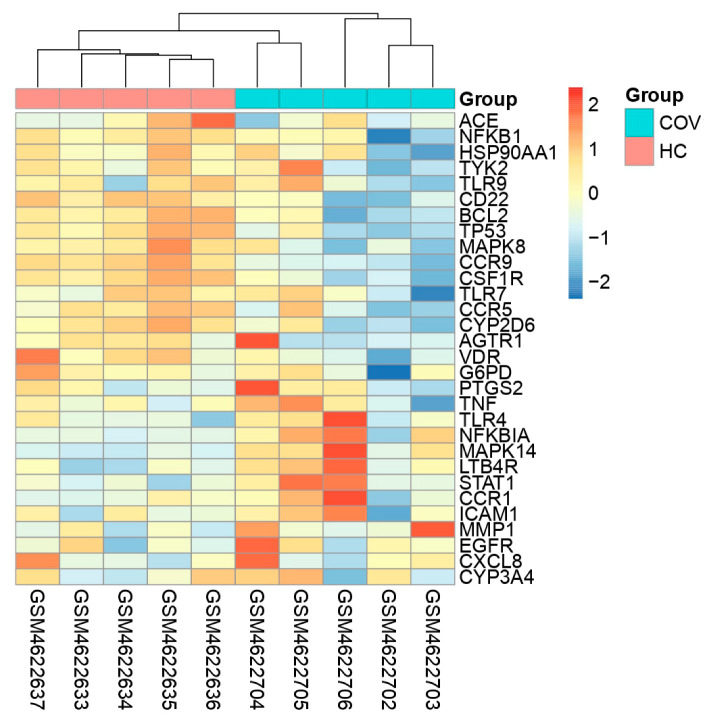
The heatmap plot denoting the 30 COVID-19-associated unique genes that are differentially regulated between the COVID-19-infected patients and healthy controls. The colored scale bar at the right indicates relative expression, where −2 and 2 represent down and up-regulation, respectively. Red color—up-regulation; blue color—down-regulation; yellow color—stable/non-significant expression. COV—COVID-19; HC—Healthy Control.

**Figure 5:**
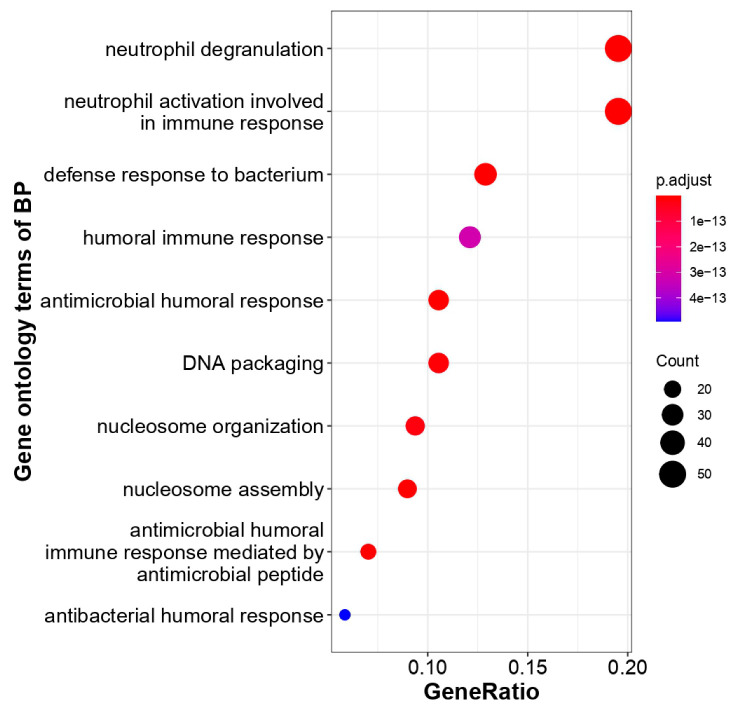
GO enrichments of COVID-19-associated unique genes with their biological processes. The number of unique COVID-19-associated genes falling in each GO biological process term is directly proportional to the count ball size. The balls are color-shaded according to the significant enrichment level (log2FC cutoff ≥ 1, *p*-value cutoff ≤ 0.05, dysregulation type—all, GO-*p*-value cutoff ≤ 0.1, GO-*q*-value cutoff ≤ 0.3).

**Figure 6:**
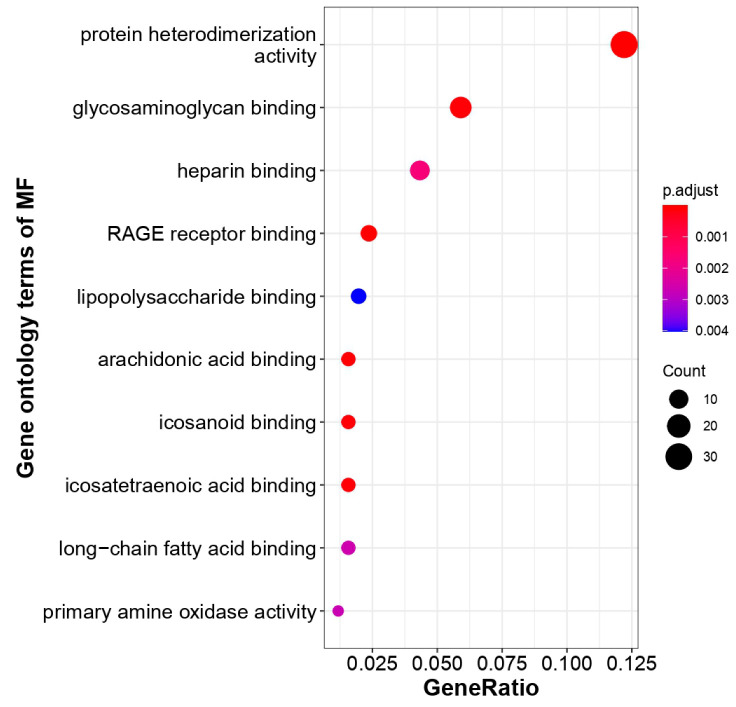
COVID-19-associated unique genes and their enriched molecular function. These unique genes’ fall in each GO molecular function term is directly proportional to the count ball size. The balls are color-shaded according to the significant enrichment level (log2FC cutoff ≥ 1, *p*-value cutoff ≤ 0.05, dysregulation type—all, GO-*p*-value cutoff ≤ 0.1, GO-*q*-value cutoff ≤ 0.3).

The unique genes and their expression profiling were imputed from COVID19db. The heatmap plot exhibits that 30 unique genes were differentially regulated and distinguishes the COVID-19-infected patients from the healthy controls (Figure 4). Further, the heatmap plot indicates the diverse physiological and pathological conditions of these unique genes. Molecular gene enrichment of these 30 COVID-19-associated unique genes were involved in various biological processes and molecular functions. These players were predicted to be involved in the significant biological regulation of nucleosome organization, humoral immune response, defense response, anti-microbial response, and neutrophil degranulation, etc. (Figure 5). They were also predicted to be involved in different molecular functions such as RAGE receptor, glycosaminoglycan, heparin, icosanoid, long-chain fatty acid binding activities, etc. (Figure 6).

### 3.7. Analysis of KEGG Pathways

**Figure 7:**
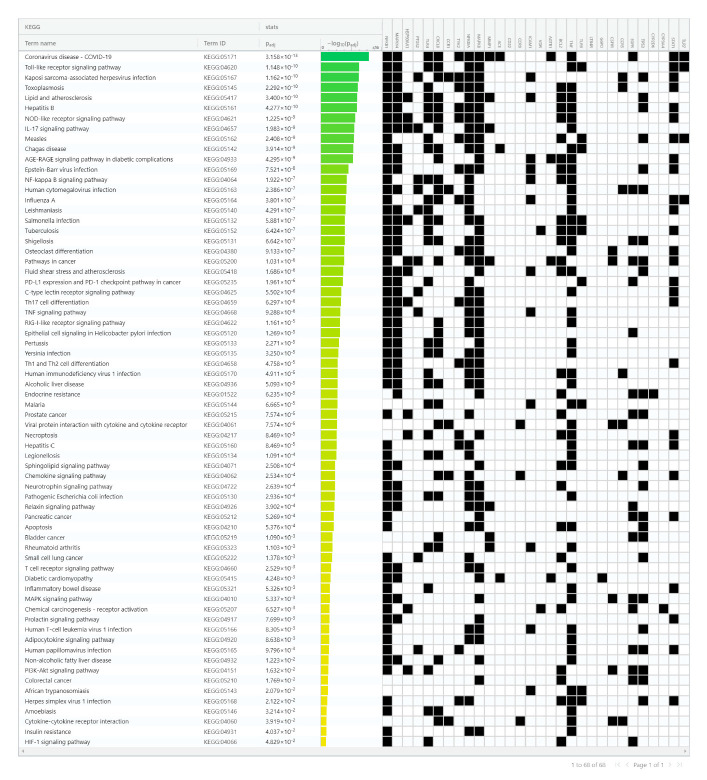
Biological pathway enrichment analysis of identified bioactives against COVID-19. KEGG enrichment pathway analysis identification result according to an adjusted *p*-value of 0.05 and the ordinate indicates the −log10 (adjusted *p*-value) of the KEGG terms.

A total of 68 KEGG pathway terms were recognized. These pathways were ranked by adjusted *p*-value—0.05 and the enrichment conditions of COVID-19-linked 30 unique genes are represented in Figure 7. These genes were predominantly involved in COVID-19, toll-like receptor signaling pathway, NOD-like receptor signaling pathway, IL-17 signaling pathway, AGE–RAGE signaling pathway in diabetic complications, NF-kappa B signaling pathway, TNF signaling pathway, MAPK signaling pathway, pathways in cancer, and so on (Figure 7). Further, the results revealed that enriched pathways of the bioactives against COVID-19 were associated with cancers, various immune responses, and other cell cycle processes. The pathway results suggested that identified bioactives may strongly perform an anti-viral effect via regulating these pathways related to COVID-19-associated targets as exposed.

### 3.8. Correlationship Expression Analysis

**Figure 8:**
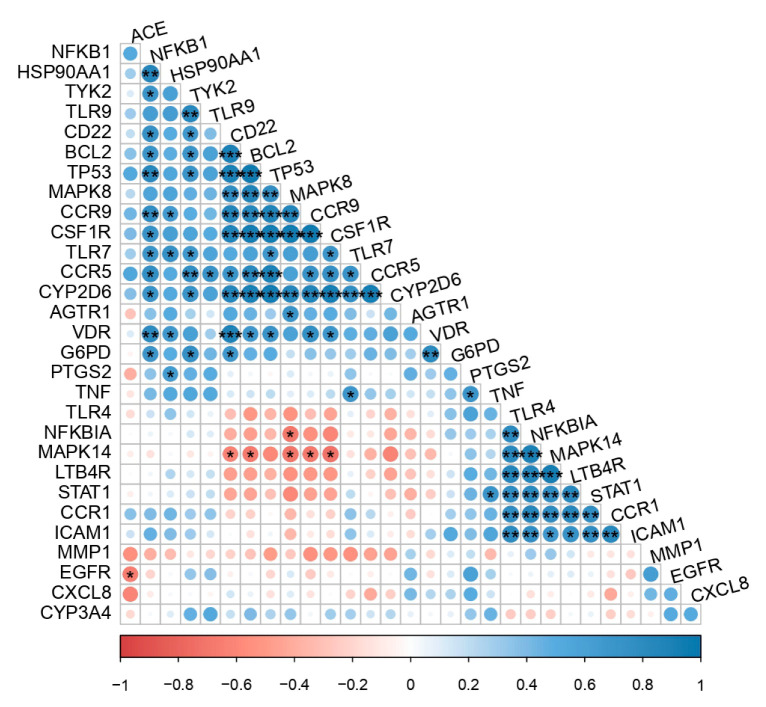
Corrplot indicates the correlationships between the unique COVID-19-associated genes. Blue color—positive correlations, red color—negative correlation, blank/white boxes—insignificant. The size of the circle and the color intensity are directly proportional to the correlation coefficients. In addition, *p*-value cutoff (*: *p* ≤ 0.05; **: *p* ≤ 0.01; ***: *p* ≤ 0.001) is to set a threshold for the significant correlation.

The COVID-19-associated unique genes and their co-expression correlationship profiling were imputed by COVID19db. Corrplot showed that 30 unique genes are the closely associated genes that have a significant correlation with each other (Figure 8). Further, the association of this immune profile might be the significant immune deregulator mechanism induced by deadly COVID-19 infection, but further validation through in vitro and in vivo is a pre-requisite.

### 3.9. Box and Volcano Plot Analysis of Unique SARS-CoV-2-Associated Genes

**Figure 9:**
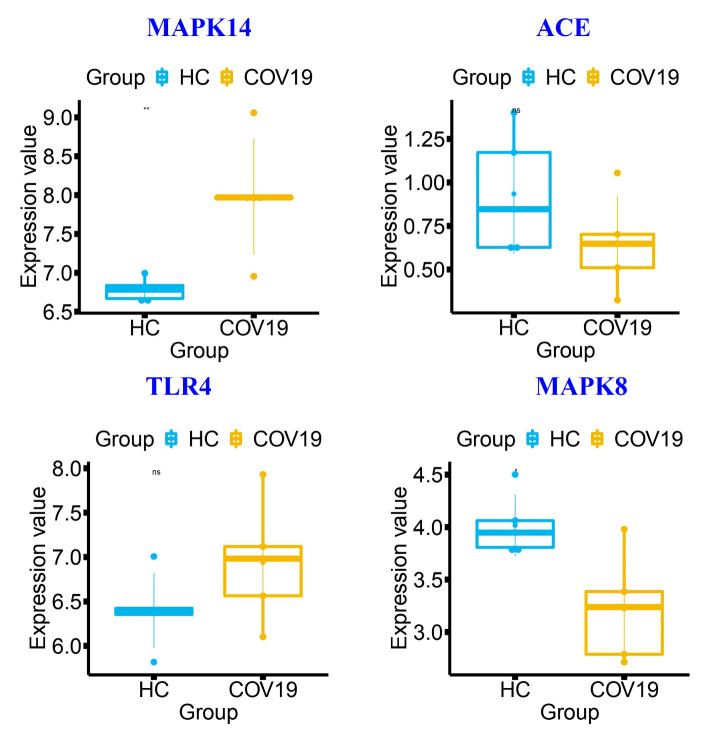
Box plots encode the 4 COVID-19-associated gene expression levels in COVID-19-infected compared with healthy controls (HCs). The plots indicate that the players were dysregulated after the SARS-CoV-2 infection. HCs are marked in blue, and COVID-19-infected tissues are marked in yellow. Significant difference of the expression value was calculated by inbuilt ANOVA and significant **: *p* ≤ 0.01; ns: *p* ≥ 0.05; *: *p* ≤ 0.05 values.

**Figure 10:**
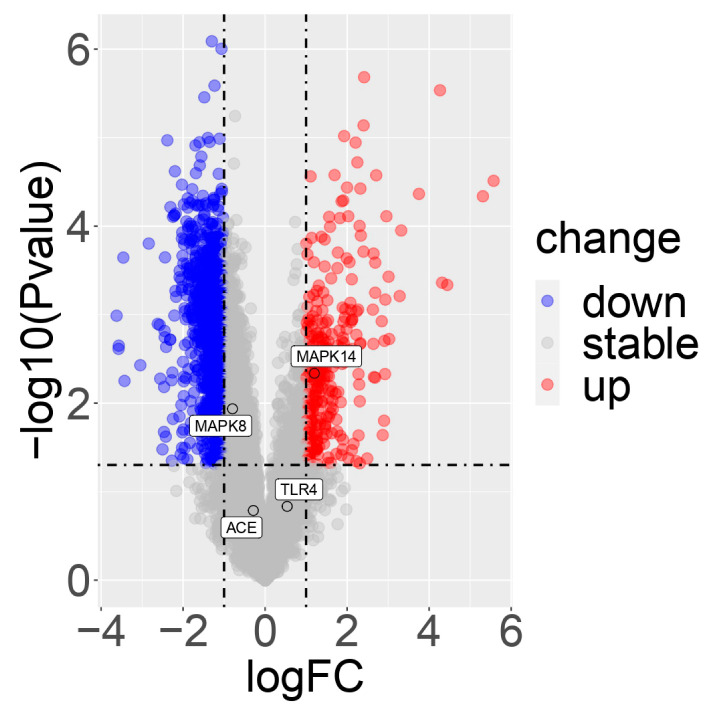
Volcano plots encode the 4 COVID-19-associated gene expression levels in COVID-19-infected whole blood tissue transcriptome datasets. Scattered balls encode genes. The x-axis indicates the log2 fold change levels between HC and COVID-19-infected, whereas the y-axis is adjusted *p*-value based on –log10. The plots indicate that the players were dysregulated after the SARS-CoV-2 infection. Red color—up-regulation, blue color—down-regulation, gray color—stable/non-significant. Genes revealed the differentially expressed players in accordance with significant thresholds.

Mitogen-activated protein kinase 14 (*MAPK14*), angiotensin-converting enzyme (*ACE*), toll-like receptor (*TLR4*), and *MAPK8* are the main players that are directly linked to the majority of the phytocompounds and played a major role in COVID-19-associated pathways. Box and volcano plots were performed for these players and suggested differential expression between different physiological and pathological conditions. Both box (Figure 9) and volcano (Figure 10) plots represent that the *MAPK14, ACE, TLR4,* and *MAPK8* genes were dysregulated after the COVID-19 infection.

### 3.10. Active Compound Property

Properties such as nVio, GPCR, Ncr, Ki, Pi, and Ei of these 25 compounds were identified using the molinspiration tool (Table 3). Phytocompounds that had zero nVio scores were considered highly significant compounds. This analysis identifies 14 compounds as significant with zero nVio score and only these compounds were employed for further analysis.

### 3.11. Molecular Docking Analysis

**Figure 11:**
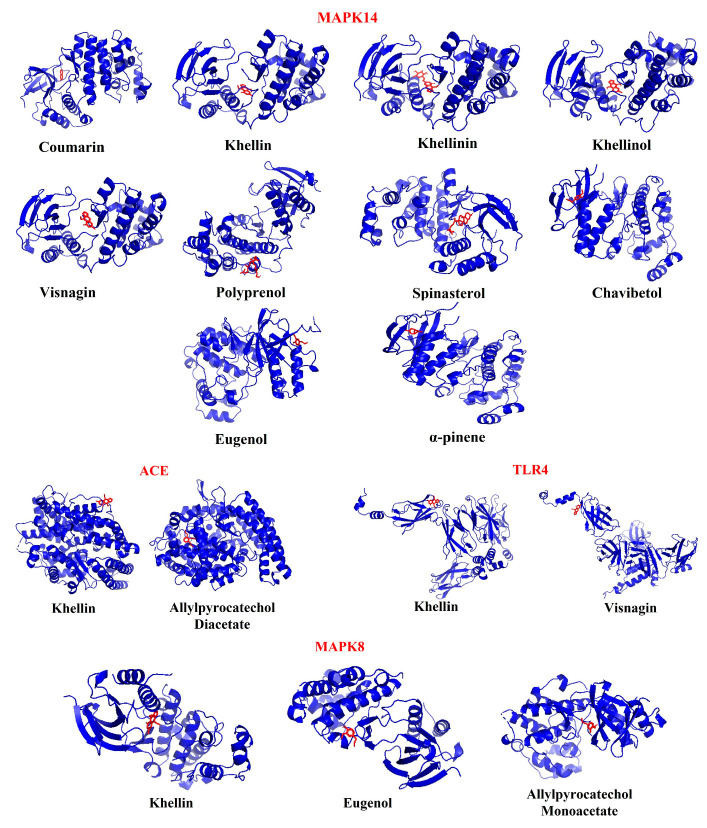
Molecular docking analysis: three-dimensional (3D) representation of interaction patterns of potentially bioactive compounds and COVID-19-associated MAPK14, ACE, TLR4, MAPK8 genes.

MAPK14, ACE, TLR4, and MAPK8 genes are present in all the compounds, and these genes are associated with COVID-19 pathways. Molecular docking was performed for these common genes with their potentially targeting compounds (Figure 11). The results are given in Table 4.

### 3.12. Identification of Significant Medicinal Plant

Molecular docking analysis revealed that the compound khellin interacts with all four COVID-19-responsible genes with significant binding energy and compounds, namely, coumarin, khellinin, khellinol, and visnagin possess significant binding energy with the COVID-19-responsible gene *MAPK14*. These compounds were present in the plant *A. visnaga* (toothpick-plant). Hence, this plant was predicted as a significant medicinal plant and its bioactives can be utilized in the treatment of COVID-19.

### 3.13. Analysis of Toxicity and Drug Likeliness Properties

The toxicity properties for the significant phytocompounds selected through molecular docking analysis were carried out using AdmetSAR and SwissADME software. Analyzing the toxicity characteristics of phytocompounds is highly essential for it to qualify as a potential drug candidate in the human body. The hERG I and II inhibition action, LD50 (rat), skin toxicity, and carcinogenicity properties of these compounds were determined using AdmetSAR software. Solubility and drug likeliness properties (Lipinski’s rule of 5) were determined using SwissADME software. The results of these analyses are tabulated in Supplementary Table S4.

## 4. Discussion

COVID-19 is a viral pandemic that predominantly affects the lungs [36] and requires comprehensive research to identify a global treatment method. Since modern medicines cannot control this disease, the search for an efficient alternative medicinal source becomes highly important. Alternative medicines are becoming a trend all over the world and, at present, nearly more than half of the world population has started using alternative medicines [37]. Plants form the basis of various successful formulations to combat various illnesses, but the mode of action and molecular mechanisms remain not fully classified [38]. Thus, this study utilizes the new arena in the name of transcriptomics, cheminformatics, and systems pharmacology to identify potential bioactives from medicinal plants and uncovers the compound associated with human COVID-19 immune targets for treating COVID-19 disease. In addition, it helps to predict the activity of these compounds in the molecular aspects. Moreover, the plant-derived bioactives are less toxic compared to synthetic drug molecules. Therefore, the current findings prescribe the bioactive molecules used to combat SARS-CoV-2 by stimulating the immune regulations.

In the present study, four medicinal plants predominantly used in India were selected and 34 bioactives were identified from the selected plants. Among 34 compounds, 25 compounds were found to possess strong interactions with 30 unique and COVID-19-associated human receptors. Subsequently, the network was constructed using Cytoscape 3.8.2 to predict the therapeutical potentials of these compounds.

In our previous study, we highlighted that multiple genes and pathways are involved in disease progression, so targeting an individual gene does not provide relevant results [10]. Hence, targeting multiple receptors at the same time might provide an efficient therapeutical effect. In this respect, the C-T-N analysis revealed multiple targets of the selected compounds. Since numerous molecular and biological factors are involved in COVID-19 disease, identifying compounds with multiple targets can provide a better solution.

Transcriptomic analyses were performed to understand the differential expression pattern of unique genes between COVID-19-infected patients and healthy controls by using publicly available transcriptomic datasets in COVID19db. A heatmap plot was generated based on the transcriptomic datasets’ (GEO accessions) expression intensities of the 30 COVID-19-associated unique genes that were differentially expressed and clearly distinguished the COVID-19-infected patients from their controls. Notably, differential expression and co-expression of these players were imputed, and they are involved in various molecular activities against COVID-19. Functional regulations and attributes of these genes were demonstrated by gene and pathway enrichments, corrplot analysis, and box and volcano plots. The results of differential expression and co-expression analyses delineate that these unique genes are more specific to COVID-19. In addition, the study showed differential expression regulation of these unique genes between different physiological and pathological conditions.

Interestingly, transcriptomic analyses confirm that these unique COVID-19 players are linked with many other viral infections such as hepatitis B and C, pertussis, influenza A, measles, tuberculosis, human papilloma virus, rheumatoid arthritis, herpes simplex virus infection, and so on. The correlation between the host immune responses to these viral infections and COVID-19 is far from clear. Suppositionally, a spectrum of drugs is already available for treating these viral infections and may have therapeutic properties against COVID-19 infection [1,39,40].

As described in KEGG enrichment analysis, these 30 unique genes were putatively involved in the regulation of various immune-response-associated pathways such as NOD-like receptor, NF-kappa B, toll-like receptor, HIF-1 signaling, IL-17, TNF, PI3K-Akt, T-cell receptor, and MAPK signaling pathways. In addition, identified bioactive compounds that target the COVID-19-associated unique genes and also inhibit their functions and their results, altering the above-mentioned pathways, and inducing the production of anti-COVID-19 antibodies through antigen-presenting cells.

Further, the pharmacological features, such as GPCR, Pi, Ki, Ncr, Ei, and nVio influenced the oral bioavailability, solubility, and permeability of the drug and were predicted through experimentally validated computational approaches in accordance with rule-of-5 (Ro5) drug discovery. Compounds that satisfied the decided criteria were selected for further studies [41,42]. Targets of those compounds were compared with the COVID-19-specific targets to identify the potential genes and were subjected to molecular docking analysis.

Finally, molecular docking analysis identified *A. visnaga* as a highly significant medicinal plant to treat COVID-19, which houses compounds such as khellin, khellinin, khellinol, and visnagin. Among the compounds, khellin interacts with all of the selected genes (*MAPK14, ACE, TLR4*, and *MAPK8*), and Khellinin interacts with *MAPK14* with the highest binding affinity of -7.9 kcal/mol. These compounds may exert potent pharmacological activity to combat SARS-CoV2 through associated immune-modulation, anti-inflammatory, and anti-viral effects. Employing the molecular docking and systems network pharmacological strategies to uncover the molecular mechanisms, anti-SARS-CoV2/COVID-19 effects of these potential bioactive molecules could be shown to be altered by key bioactives and some corresponding genes such as *MAPK14, ACE, TLR4*, and *MAPK8*. These are significant genes that are directly correlated with COVID-19 infection [1,20,21,22]. In general, *MAPK14* is one of the important components of the MAP kinase signaling pathway, which activates the transcription factors directly when evoked by physical stress or cytokines. *MAPK8* is also involved in several activities similar to *MAPK14* and also in various other cellular processes such as apoptosis, proliferation, and differentiation. *TLR4* is important in innate immune response mechanisms against antigens and stimulates pro-inflammatory responses. Consequently, an interaction between TLR4 and SARS-CoV2 spike proteins could be the reason behind the COVID-19 immunopathological expression [1]. The activity of ACE leads to an increase in vasoconstriction by inactivating the vasodilator agent, bradykinesia. In addition, the main cellular receptor for SARS-CoV2 is ACE, the expression of ACE genes (ACE1 and ACE2), which induce exposure to infection. Further, this gene has been associated with respiratory tract disease and plays a prominent role in the severity of SARS-CoV2 [43]. Toxicity and drug likeliness analysis results made evident that none of the selected phytocompounds possess toxicity properties. These compounds do not violate the Lipinski rule of 5, which clears the primary evaluation for their drug likeliness activity. Further, solubility is one of the significant parameters to determine the pharmacological response of the drug [44] and all the selected compounds were found to be soluble. Thus, these compounds can be considered as a potential drug candidate, but the imputed results are still required to be experimentally validated rigorously. These results claim that bioactive compounds in *A. visnaga* plants have dynamic interactions with COVID-19-responsible genes, thus possessing significant therapeutical actions.

## 5. Conclusions

The present study reveals the therapeutic potentials of commonly used Indian medicinal plants against the deadly COVID-19 pandemic. In short, amidst the selected four medicinal plants, *A. visnaga*, commonly known as toothpick-plant, was predicted as a potent medicinal plant against COVID-19. Bioactive compounds in this plant target the significant COVID-19 responsible receptors such as MAPK14, ACE, TLR4, and MAPK8, which makes this plant an ideal candidate for treating this deadly infection. Traditionally, this plant has multiple medicinal properties and is generally prescribed in Ayurvedic formulations to dilate blood vessels without affecting blood pressure. In some parts of the world, this plant was used against kidney and heart ailments. In a line, this study explores the possibility of the usage of *A. visnaga* in COVID-19 treatment and also unravels the various biological processes and their signaling interactions that will aid the way to open the COVID-19 advanced research sluicegates in a combination of Indian Ayurveda and modern medicine. Further, the study also serves as a notable pioneer for researchers and budding scientists working in the era of medicinal plant biology/omics by employing systems pharmacology approaches.

Further, this pilot study hypothesizes that the formulation of phytocompounds present in *A. visnaga* can be used in the treatment of COVID-19 disease. However, these predictions need to be evaluated using in vitro and in vivo experimentations to design antagonists for COVID-19-responsible human targets, which ultimately may result in the cure of COVID-19 disease. In addition, this study undoubtedly provides a conceptual shift in the development of drug discovery and creates an impact on the globalization and modernization of plant bioactive molecules. Our holistic study lays the groundwork for enabling further research on the molecular perspective of Indian traditional medicinal plants in disease treatment, immunobiology of various diseases including SARS-CoV2, and the applications of systems pharmacology in novel drug discovery.

## Figures and Tables

**Table 1:** List of phytocompounds with their abbreviations.

S.No.	Common Name	Scientific Name	Compounds	Abbreviations
1	Toothpick-plant	*A. visnaga*	γ-pyrones (furanochromone)	PYR
			Khellin	KHE
			Visnagin	VGN
			Khellinol	KHNOL
			Ammiol	AMM
			Khellol	KLOL
			Khellinin	KHEL
			Coumarin	CM
2	Slender amaranth	*A. viridis*	Quercetin	QUE
			Lutein	LUE
			Rutin	RU
			Beta carotene	BCAR
			Amasterol	AMST
			Squalene	SQU
			Spinasterol	SPIN
			Polyprenol	POPRE
			Phytol	PHY
3	Bibhitaki	*T. bellirica*	Corilagin	COR
			Chebulagic acid	CHA
			Galloylpunicalagin	GALLO
			Cardenolides	CARD
4	Betel leafs	*P. betle*	Chavibetal	CHAVI
			Caryophyllene	CAPH
			Chavibetol acetate	CHAVIA
			Allylpyrocatechol Diacetate	ALYD
			Chavibetal methyl ether	CHAVIME
			Campene	CAPE
			β-Pinene	βPIN
			Eugenol	EU
			u-limonene	LIME
			α-Pinene	αPIN
			1,8-Cineol	CIN
			Saprobe	SPRO
			Allylpyrocatechol Monoacetate	ALYM

**Table 2:** Features of compound-targeted potential receptors.

S.No.	Compound	Target	UniProt ID	Chr. No.	Orthologs
1	Pyrones	*NFKB1*	P19838	4	*Nfkb1*
2	Khellin	*HSP90AA1*	P07900	14	*Hsp90aa1*
3	Visnagin	*PTGS2*	Q6ZYK7	1	*Ptgs2*
4	Khellinol	*PTGS2*	Q6ZYK7	1	*Ptgs2*
5	Khellinin	*MMP1*	P03956	11	*Mmp1a*
6	Coumarin	*NFKB1*	P19838	4	*Nfkb1*
7	Quercetin	*EGFR*	Q504U8	7	*Egfr*
8	Lutein	*VDR*	P11473	12	*Vdr*
9	Rutin	*PTGS2*	Q6ZYK7	1	*Ptgs2*
10	Beta carotene	*MAPK14*	Q16539	6	*Mapk14*
11	Amasterol	*VDR*	P11473	12	*Vdr*
12	Squalene	*CCR5*	P51681	3	*Ccr5*
13	Spinasterol	*VDR*	P11473	12	*Vdr*
14	Polyprenol	*VDR*	P11473	12	*Vdr*
15	Phytol	*CCR1*	P32246	3	*Ccr1l1*
16	Corilagin	*PTGS2*	Q6ZYK7	1	*Ptgs2*
17	Chebulagic acid	*PTGS2*	Q6ZYK7	1	*Ptgs2*
18	Cardenolides	*MAPK14*	Q16539	6	*Mapk14*
19	Chavibetal	*MAPK14*	Q16539	6	*Mapk14*
20	Caryophyllene	*CCR5*	P51681	3	*Ccr5*
21	Allylpyrocatechol Diacetate	*TYK2*	P29597	19	*Tyk2*
22	Eugenol	*TLR7*	Q9NYK1	X	*Tlr7*
23	α-Pinene	*MAPK14*	Q16539	6	*Mapk14*
24	1,8-Cineol	*HSP90AA1*	P07900	14	*Hsp90aa1*
25	Allylpyrocatechol Monoacetate	*TLR7*	Q9NYK1	X	*Tlr7*

**Table 3:** Bio-properties of phytocompounds.

S. No.	Compounds	GPCR Ligand	Kinase Inhibitor	Nuclear Receptor Ligand	Protease Inhibitor	Enzyme Inhibitor	No. of Violations
1	Pyrones	−0.63	−0.95	−1.12	−1.28	−0.23	0
2	Khellin	−0.36	−0.51	−0.51	−0.64	−0.07	0
3	Visnagin	−0.55	−0.79	−0.79	−0.92	−0.28	0
4	Khellinol	−0.39	−0.64	−0.49	−0.77	0.01	0
5	Khellinin	0.07	−0.23	−0.37	−0.06	0.29	0
6	Coumarin	−1.44	−1.57	−1.42	−1.43	−0.58	0
7	Quercetin	−0.06	0.28	0.36	−0.25	0.28	0
8	Lutein	0.03	−0.25	0.47	−0.03	0.28	2
9	Rutin	−0.05	−0.14	−0.23	−0.07	0.12	3
10	Beta carotene	−0.04	−0.15	0.4	−0.06	0.17	2
11	Amasterol	0.09	−0.31	0.88	−0.14	0.61	1
12	Squalene	0.04	−0.1	0.19	−0.03	0.16	1
13	Spinasterol	0.18	−0.3	0.68	0.06	0.53	1
14	Polyprenol	0.14	−0.14	0.37	0.02	0.33	1
15	Phytol	0.11	−0.32	0.35	0	0.31	1
16	Corilagin	−0.11	−0.45	−0.44	−0.03	−0.15	3
17	Chebulagic acid	−3.5	−3.72	−3.68	−3.23	−3.56	3
18	Cardenolides	0.11	−0.48	0.39	−0.17	0.63	0
19	Chavibetal	−0.86	−1.14	−0.78	−1.29	−0.41	0
20	Caryophyllene	−0.34	−0.78	0.13	−0.6	0.19	1
21	Allylpyrocatechol Diacetate	−0.46	−0.75	−0.25	−0.67	−0.18	0
22	Eugenol	−0.86	−1.14	−0.78	−1.29	−0.41	0
23	α-Pinene	−0.48	−1.5	−0.62	−0.85	−0.34	0
24	1,8-Cineol	−0.93	−1.6	−1.07	−0.9	−0.15	0
25	Allylpyrocatechol Monoacetate	−0.7	−1.04	−0.39	−0.95	−0.23	0

**Table 4:** The binding affinity of compounds docked against human target receptors.

Receptor	Ligand	Binding Affinity (kcal/mol)
MAPK14	Coumarin	−6.1
	Khellin	−6.1
	Khellinin	−7.9
	Khellinol	−7
	Visnagin	−6.8
	Polyprenol	−2.8
	Spinasterol	−6
	Chavibetol	−5.9
	Eugenol	−5.8
	α-pinene	−6
ACE	Allylpyrocatechol Diacetate	−5.5
	Khellin	−6.4
TLR4	Khellin	−5.7
	Visnagin	−6.3
MAPK8	Khellin	−6.4
	Allylpyrocatechol Monoacetate	−5.5
	Eugenol	−5.3

## Data Availability

All data generated or analyzed during this study are included in this article and its Supplementary Information files.

## Supplementary Materials

The following supporting information can be downloaded at: https://www.mdpi.com/article/10.3390/molecules27185955/s1, Table S1: List of COVID-19-associated genes collected from the literature; Table S2: Bioactive compounds and their canonical SMILES and structures; Table S3: Identified 30 unique/common genes associated with COVID-19; Table S4: Toxicity and drug likeliness properties of potential compounds

## Sample Availability

Samples of the compounds are not available from the authors.

## Abbreviations

COVID-19	Coronavirus disease-19
C-T-N	Compound-target-network
ORA	Over-representation analysis
WHO	World Health Organization
SARS-CoV2	Severe acute respiratory syndrome coronavirus 2
