# Supplementary material for: Transcriptomics, Cheminformatics, and Systems Pharmacology Strategies Unveil the Potential Bioactives to Combat COVID-19
Source: Molecules. 2022 Sep 13;27(18):5955. doi: 10.3390/molecules27185955 (PMC9503185; doi:10.3390/molecules27185955)
Supplement: Supplementary file 1 [file molecules-27-05955-s001.zip › Supplementary Table S1.pdf]

molecules

MDPI

Supplementary

Transcriptomics, Cheminformatics, and Systems Pharmacology Strategies Unveil the Potential Bioactives to Combat COVID-19

Sivakumar Adarshan[1], Sakthivel Akassh[2†], Krishnakumar Avinash[2†], Mathivanan Bharathkumar[2†], Pandiyan Muthuramalingam[2,3,4*], Hyunsuk Shin[3,4*], Venkidasamy Baskar[5], Jen-Tsung Chen[6*], Veluswamy Bhuvaneshwari[7] and Manikandan Ramesh[1]

Table S1: List of COVID-19 associated genes collected from the literature

S.No.	Genes	S.No.	Genes	S.No.	Genes	S.No.	Genes
1	ACE1	36	CYP3A4	71	ACE	106	*LCN2,*
2	ACE2	37	GSTM1	72	AT1R	107	STAT1
3	ABO	38	GSTP1	73	ABCB	108	UBE2L6
4	SRY	39	HAMP	74	SLC6A20	109	RDRP
5	SOX3	40	HLA-B	75	ERMP1	110	HA
6	ADAM17	41	HLA-DRB1	76	FCER1G	111	HLA
7	TMPRSS2	42	IFNA1	77	CA11	112	*LZTFL1*
8	EGFR	43	MMP1	78	IFNAR2	113	FYCO1
9	FN1	44	SERPINA1	79	TYK2	114	CXCR6
10	HSP90AA1	45	TIMP2	80	DPP9	115	XCR1
11	TP53	46	RPA2	81	CCR2.	116	*IL1B*
12	CALM1	47	POLD4	82	TLR	117	IL1R1
13	MAST2	48	MAPK8	83	TLR4	118	IL1RN
14	PTGS2	49	IRF7	84	STAT1	119	IL17A
15	TNF	50	JUN	85	SELL	120	FCGR2A
16	IL6	51	NFKB1	86	PSMB9	121	IRF7
17	TLR3	52	NFKBIA	87	CD22	122	JUN
18	TLR7	53	CD40LG	88	CCR1	123	FASLG
19	TLR8	54	FASLG	89	CCR5	124	ICAM1
20	TLR9	55	ICAM1	90	LTB4R	125	LIFR
21	ITPA	56	LIFR	91	MAPK14	126	STAT2
22	IFITM3	57	STAT2	92	CSF1R	127	IL-8
23	LZTFL1	58	CCR1	93	BCL2	128	CXCL10
24	SLC6A20	59	G6PD	94	CASP1	129	IL-1β
25	IL10RB	60	IFIT1	95	NLRP3	130	M-CSF
26	IFNAR2	61	OAS2	96	UQCRH		
27	OAS1	62	OAS3	97	PPA2		

28	CCR9	63	OASL	98	PAPSS1
29	EGF	64	IL28B	99	AP006621.5
30	CSF2	65	VDR	100	CMB9-55F22.1
31	CXCL8	66	SLCO1B3	101	AP006621.6
32	AGTR1	67	ABCC2	102	PANOI
33	CCL2	68	LEP	103	CTD-2027I19.2
34	CFH	69	CETP	104	LINC01273
35	CYP2D6	70	MCP-1	105	ARSA
