# Supplementary material for: Transcriptomics, Cheminformatics, and Systems Pharmacology Strategies Unveil the Potential Bioactives to Combat COVID-19
Source: Molecules. 2022 Sep 13;27(18):5955. doi: 10.3390/molecules27185955 (PMC9503185; doi:10.3390/molecules27185955)
Supplement: Supplementary file 1 [file molecules-27-05955-s001.zip › Supplementary Table S2.pdf]

molecules

Supplementary

Transcriptomics, Cheminformatics, and Systems Pharmacology Strategies Unveil the Potential Bioactives to Combat COVID-19

Sivakumar Adarshan[1], Sakthivel Akassh[2†], Krishnakumar Avinash[2†], Mathivanan Bharathkumar[2†], Pandiyan Muthuramalingam[2,3,4*], Hyunsuk Shin[3,4*], Venkidasamy Baskar[5], Jen-Tsung Chen[6*], Veluswamy Bhuvaneshwari[7] and Manikandan Ramesh[1]

Table S2: Bioactive compounds and their canonical SMILES and structures.

S.No.	Compounds	Canonical SMILES	Structure
1	PYR	C1=COC2=CC3=C(C=C21)C(=O)C=CO3	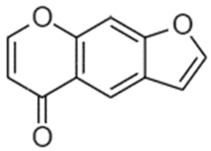
2	KHE	CC1=CC(=O)C2=C(C3=C(C(=C2O1)OC)OC=C3)OC	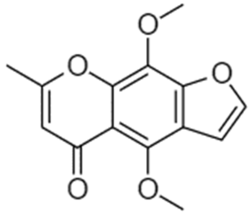
3	VGN	CC1=CC(=O)C2=C(O1)C=C3C(=C2OC)C=CO3	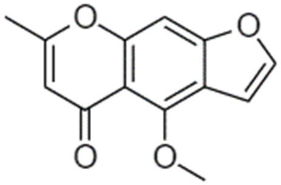

4	KHNOL	CC1=CC(=O)C2=C(O1)C(=C3C(=C2O)C=CO3)OC	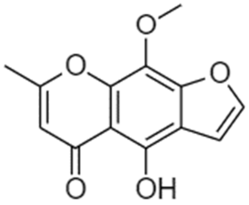
5	AMM	COC1=C2C(=O)C=C(OC2=C(C3=C1C=CO3)OC)CO	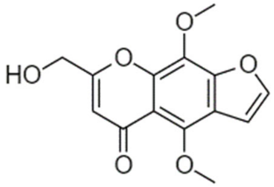
6	KLOL	COC1=C2C=COC2=CC3=C1C(=O)C=C(O3)CO	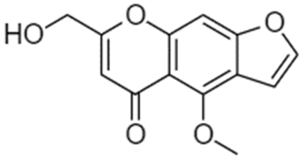
7	KHEL	COC1=C2C=COC2=CC3=C1C(=O)C=C(O3)CO[C@H]4[C@@H]([C@H]([C@@H]([C@H](O4)CO)O)O)O	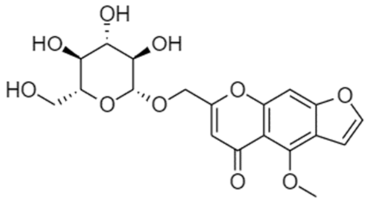
8	CM	C1=CC=C2C(=C1)C=CC(=O)O2	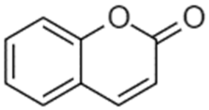
9	QUE	C1=CC(=C(C=C1C2=C(C(=O)C3=C(C=C(C=C3O2)O)O)O)O)O	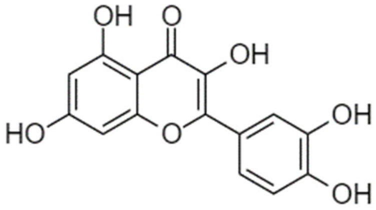

molecules MDPI

10	LUE	CC1=C(C(CC(C1)O)(C)C)C=CC(=CC=CC(=CC=CC=C(C)C=CC=C(C)C=CC2C(=CC(CC2(C)C)O)C)C)C	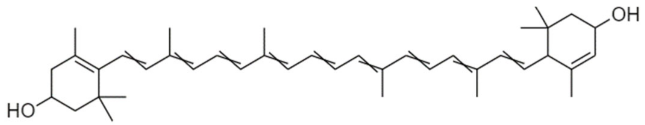
11	RU	CC1C(C(C(C(O1)OCC2C(C(C(C(O2)OC3=C(OC4=CC(=CC(=C4C3=O)O)O)C5=CC(=C(C=C5)O)O)O)O)O)O)O)O	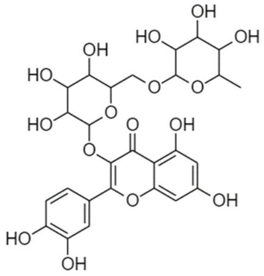
12	BCAR	CC1=C(C(CCC1)(C)C)C=CC(=CC=CC(=CC=CC=C(C)C=CC=C(C)C=CC2=C(CCCC2(C)C)C)C)C	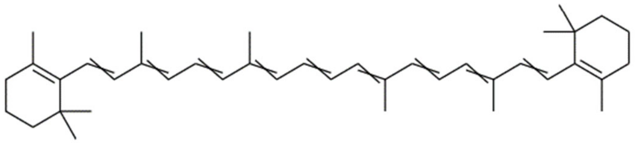
13	AMST	CC(C)C(=C)CCC(C)(C1CCC2C1(CCC3C2=CC=C4C3(CCC(C4)O)C)C)O	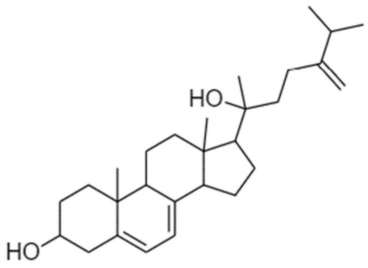
14	SQU	CC(=CCCC(=CCCC(=CCCC=C(C)CCC=C(C)CCC=C(C)C)C)C)C	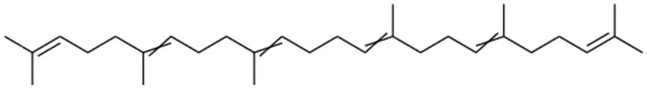

15	SPIN	CCC(C=CC(C)C1CCC2C1(CCC3C2=CCC4C3(CCC(C4)O)C)C)C(C)C	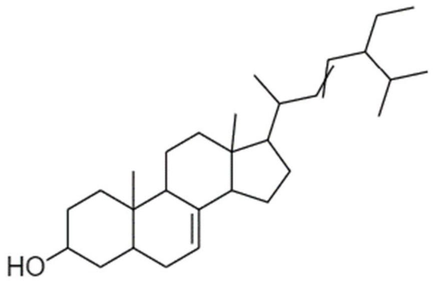
16	POPRE	CC(=CCCC(=CCCC(=CCCC(=CCCC(=CCO)C)C)C)C)C	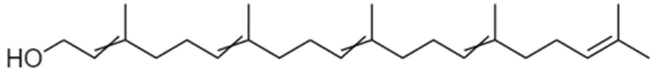
17	PHY	CC(C)CCCC(C)CCCC(C)CCCC(=CCO)C	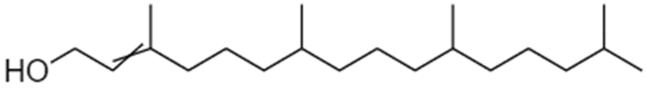
18	COR	C1C2C(C(C(C(O2)OC(=O)C3=CC(=C(C(=C3)O)O)O)O)OC(=O)C4=CC(=C(C(=C4C5=C(C(=C(C=C5C(=O)O1)O)O)O)O)O) O)O	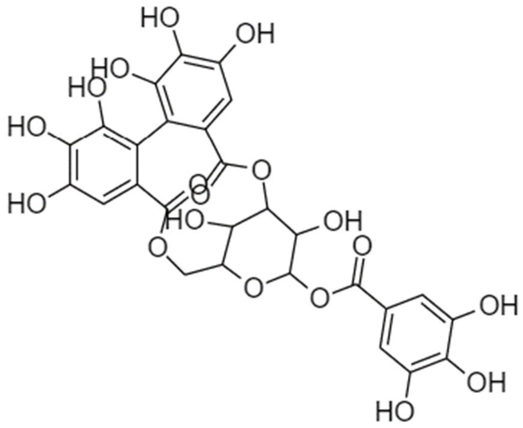

molecules

19	CHA	C1C2C3C(C(C(O2)OC(=O)C4=CC(=C(C(=C4)O)O)O)OC(=O)C5=CC(=C(C6=C5C(C(C(=O)O3)CC(=O)O)C(C(=O)O6)O)O)O)OC(=O)C7=CC(=C(C(=C7C8=C(C(=C(C=C8C(=O)O1)O)O)O)O)O)O	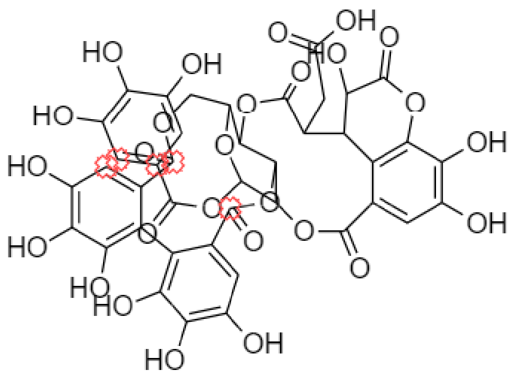
20	GALLO	NA	NA
21	CARD	CC12CCCCC1CCC3C2CCC4(C3CCC4C5=CC(=O)OC5)C	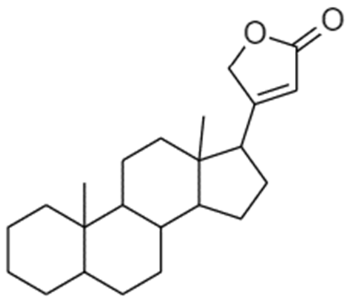
22	CHAVI	COC1=C(C=C(C=C1)CC=C)O	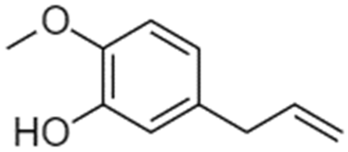
23	CAPH	CC1=CCCC(=C)C2CC(C2CC1)(C)C	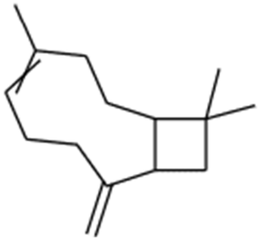

24	CHAVIA	NA	NA
25	ALYD	CC(=O)OC1=CC=CC(=C1OC(=O)C)CC=C	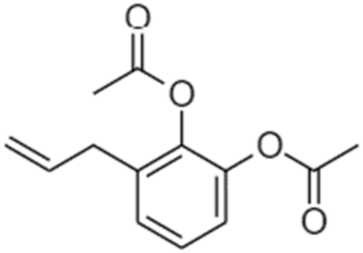
26	CHAVIME	NA	NA
27	CAPE	CC1(C2CCC(C2)C1=C)C	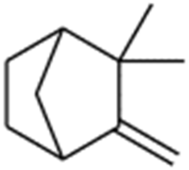
28	FPIN	NA	NA
29	EU	COC1=C(C=CC(=C1)CC=C)O	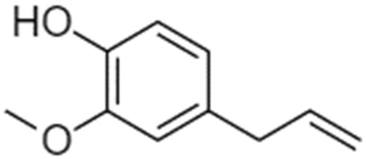
30	LIME	NA	NA
31	PIN	CC1=CCC2CC1C2(C)C	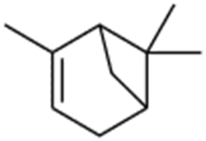
32	CIN	CC1(C2CCC(O1)(CC2)C)C	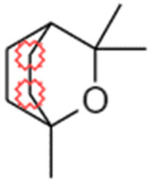
33	SPRO	NA	NA

molecules MDPI

34	ALYM	CC(=O)OC1=C(C=C(C=C1)CC=C)O	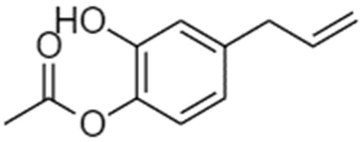

NA: Not Available
