# Supplementary material for: Transcriptomics, Cheminformatics, and Systems Pharmacology Strategies Unveil the Potential Bioactives to Combat COVID-19
Source: Molecules. 2022 Sep 13;27(18):5955. doi: 10.3390/molecules27185955 (PMC9503185; doi:10.3390/molecules27185955)
Supplement: Supplementary file 1 [file molecules-27-05955-s001.zip › Supplementary Table S3.pdf]

molecules

MDPI

Supplementary

Transcriptomics, Cheminformatics, and Systems Pharmacology Strategies Unveil the Potential Bioactives to Combat COVID-19

Sivakumar Adarshan[1], Sakthivel Akassh[2†], Krishnakumar Avinash[2†], Mathivanan Bharathkumar[2†], Pandiyan Muthuramalingam[2,3,4*], Hyunsuk Shin[3,4*], Venkidasamy Baskar[5], Jen-Tsung Chen[6*], Veluswamy Bhuvaneshwari[7] and Manikandan Ramesh[1]

Table S3: Identified 30 unique/common genes associated with COVID-19.

S.No.	Compounds	Targets
1	Coumarin	NFKB1
		EGFR
		MAPK14
2	Khellin	HSP90AA1
		PTGS2
		TLR4
		CXCL8
		CCR1
		MAPK14
		TYK2
		NFKBIA
		MAPK8
3	Khellinin	MMP1
		MAPK14
		ACE
		CD22
4	Khellinol	PTGS2
		HSP90AA1
		MAPK14
5	Visnagin	PTGS2
		MAPK14
		CXCL8
		TLR4
		CCR9
		TYK2
		CCR1
		ICAM1
6	Pyrones	NFKB1
		HSP90AA1
		CCR1

		TLR4
		ACE
		ICAM1
		MAPK14
7	Amasterol	VDR
		CCR1
		AGTR1
		CCR9
		BCL2
8	Beta carotene	MAPK14
		VDR
		TNF
9	Lutein	VDR
		TNF
		BCL2
		TLR9
		LTB4R
		G6PD
		MAPK14
10	Phytol	CCR1
		CSF1R
		CCR5
11	Polyprenol	VDR
		CCR1
		PTGS2
		EGFR
		MAPK14
		AGTR1
12	Quercetin	EGFR
13	Rutin	PTGS2
		TNF
		TP53
		EGFR
14	Spinasterol	VDR
		G6PD
		PTGS2
		CSF1R
		CCR1
		MAPK14
15	Squalene	CCR5
		VDR
		PTGS2
		EGFR

		BCL2
		AGTR1
16	Cardenolides	MAPK14
		CYP2D6
		CYP3A4
17	Chebulagic acid	PTGS2
		MAPK14
		BCL2
		STAT1
18	Corilagin	PTGS2
		HSP90AA1
		EGFR
19	Chavibetol	MAPK14
		MAPK8
		TLR9
		TLR7
20	Caryophyllene	CCR5
		VDR
21	Eugenol	TLR7
		TLR9
		MMP1
		MAPK8
		TYK2
		MAPK14
22	a-Pinene	MAPK14
		CCR5
		G6PD
23	1,8-Cineol	HSP90AA1
		VDR
24	Allylpyrocatechol Monoacetate	TLR7
		TLR9
		MAPK8
25	Allylpyrocatechol Diacetate	TYK2
		ACE
		TLR9
		EGFR
		CSF1R

These 25 compounds majorly targeting the 30 unique genes
