# Supplementary material for: Transcriptomics, Cheminformatics, and Systems Pharmacology Strategies Unveil the Potential Bioactives to Combat COVID-19
Source: Molecules. 2022 Sep 13;27(18):5955. doi: 10.3390/molecules27185955 (PMC9503185; doi:10.3390/molecules27185955)
Supplement: Supplementary file 1 [file molecules-27-05955-s001.zip › Supplementary Table S4.pdf]

molecules

Supplementary

Transcriptomics, Cheminformatics, and Systems Pharmacology Strategies Unveil the Potential Bioactives to Combat COVID-19

Sivakumar Adarshan[1], Sakthivel Akassh[2†], Krishnakumar Avinash[2†], Mathivanan Bharathkumar[2†], Pandiyan Muthuramalingam[2,3,4*], Hyunsuk Shin[3,4*], Venkidasamy Baskar[5], Jen-Tsung Chen[6*], Veluswamy Bhuvaneshwari[7] and Manikandan Ramesh[1]

Table S4: Toxicity and drug likeliness properties of potential compounds

Compound	hERG I & II inhibitor	LD50 (rat) (mol/kg)	Skin toxicity	Carcinogenicity	Lipinski rule	Solubility
KHE	No	2.186	No	No	nVio	Yes
VGN	No	2.141	No	No	nVio	Yes
KHNOL	No	1.31	No	No	nVio	Yes
KHEL	No	2.487	No	No	nVio	Yes
CM	No	2.112	No	No	nVio	Yes

nVio-No Violations
